# Biology of clinical strains of *Mycobacterium tuberculosis* with varying levels of transmission

**DOI:** 10.1016/j.tube.2018.02.003

**Published:** 2018-03

**Authors:** Crystal A. Shanley, Marcela I. Henao-Tamayo, Chand Bipin, Raja Mugasimangalam, Deepshika Verma, Diane J. Ordway, Elizabeth M. Streicher, Ian M. Orme

**Affiliations:** a Mycobacteria Research Laboratories, Department of Microbiology, Immunology and Pathology, Colorado State University, Fort Collins, CO, USA; b Genotypic Technology Ltd, Bangalore, Karnataka, India; c Biomedical Sciences, University of Stellenbosch, Tygerberg, Western Cape, South Africa

**Keywords:** Tuberculosis clinical strains, Disease transmission, Guinea pig model, Immune response, Pathogenesis

## Abstract

Transmission of *Mycobacterium tuberculosis* bacilli from one individual to another is the basis of the disease process. While considerable emphasis has been placed on the role of host mechanisms of resistance in establishing or preventing new infection, far less has been expended on understanding possible factors operative at the bacterial level. In this study we established a panel of clinical isolates of *M. tuberculosis* strains obtained from the Western Cape region of South Africa, each of which had been carefully tracked in terms of their degree of transmission in the community. Each of the panel were used to infect guinea pigs with 15–20 bacilli by aerosol exposure and the course of the infection then determined. Strains with different degrees of transmission could not be distinguished in terms of their capacity to grow in the main target organs of infected animals. However, rather surprisingly, while strains with no evidence of transmission [NOT] in general caused moderate to severe lung damage, this parameter in animals infected with highly transmitted [HT] strains was mostly mild. In terms of TH1 immunity these signals were strongest in these latter animals, as was IL-17 gene expression, whereas minimal signals for regulatory molecules including IL-10 and FoxP3 were seen across the entire panel. In terms of T cell numbers, responses of both CD4 and CD8 were both far faster and far higher in animals infected with the HT strains. At the gene expression level we observed a major three-fold difference [both up and down] between NOT and HT strains, but in terms of proteins of key interest only a few [including PD-L1 and HIF-3] showed major differences between the two groups. Overall, it was apparent that NOT strains were far more inflammatory that HT strains, and appeared to trigger a much larger number of genes, possibly explaining the observed damage to the lungs and progressive pathology. In contrast, the HT strains, while equally virulent, were more immunogenic and developed much stronger T cell responses, while keeping lung damage to a minimum. Hence, in terms of trying to explain the capacity of these strains to cause transmission, these results are clearly paradoxical.

## 1 Introduction

Tuberculosis, now the number one cause of death from an infectious disease caused by a single pathogen [1], transmitted by coughing after the bacillus is exhaled from the infected lung. As a result, the central objective of chemotherapy and/or vaccination for tuberculosis is not just to treat or prevent it, but to block transmission of the disease from one individual to another.

While the basic mechanics of transmission are understood, practical models of this event are missing, including models in which vaccination might be used to prevent infection rather than disease, a difficult issue in itself [2]. Human to guinea pig models have been developed [[3], [4], [5]] and more recently attempts have been made in terms of inter-species models – non-human primates to guinea pigs – and within single species [mixing infected and healthy cattle for example]. At this time however no new information seems to have come out of these approaches.

A further limitation here is the fact that these studies mostly use the laboratory strains for infection, usually H37Rv. While this continues to provide useful information, it must be borne in mind that studies using the newly emerging clinical strains indicate that they can behave differently, demonstrate a considerable range of virulence, can induce much broader T cell subset responses, and can be inhibited by prior BCG vaccination to different degrees [[6], [7], [8], [9], [10], [11], [12], [13], [14], [15]]. A further element of disease transmission however, which has rarely been addressed to date, is the intrinsic nature of the *M. tuberculosis* isolate itself in terms of its actual ability to be transmitted. In this regard, it is now evident that, apparently independently of which family a given isolate belongs to, certain isolates appear to undergo very substantial transmission through communities, whereas others show very little transmission, if any at all [“unique” or “no observed transmission, NOT]. This suggests that individual isolates have biological properties that affect the degree to which they can be transmitted [[16], [17], [18]]. What these properties might be is unknown, for the simple reason that while thousands of isolates have undergone molecular genotyping, only a very few have ever been tested in animal models in which the host response to these isolates can be compared.

The present study was based on collecting a library of isolates from the Western Cape of South Africa which had undergone epidemiologic genetic typing. Some of these had been widely transmitted across this region, with some isolates causing very large numbers of disease clusters/outbreaks, and with others causing very low numbers, if any. Each isolate was tested in the low dose aerosol guinea pig infection model, and the pathologic and immunologic host response then measured against time using conventional assays.

Several of our observations were surprising, and not easy to explain. No major differences were seen between any of the isolates in terms of their ability to grow in the guinea pig lungs. However, contrary to expectations, NOT strains gave rise to much more extensive lung damage compared to HT strains. In contrast, the latter appeared far more immunogenic, with stronger and more rapid T cell responses. Finally, infection with NOT strains increased the degree of expression of three-fold more genes in the lungs compared to HT strains. How these overall results might pertain to their transmission patterns is not resolved by these findings, but does suggest some clues, which are discussed.

## 2 Methods

### 2.1 Guinea pigs

Specific pathogen free, female outbred Hartley guinea pigs (∼450–500 g in weight) were purchased from the Charles River Laboratories (North Wilmington, MA) and held under barrier conditions in a biosafety level III animal laboratory. The specific-pathogen-free nature of the guinea pig colonies was demonstrated by testing sentinel animals. All experimental protocols were approved by the Animal Care and Usage Committee of Colorado State University and comply with NIH guidelines. Prior to *M. tuberculosis* challenge, animals were appropriately acclimatized, then microchipped for individual animal identification [19].

### 2.2 Experimental infections

All strains used in this study were collected in the Western Cape region of South Africa. These strains originated from patients from a suburb in Cape Town with a high burden of tuberculosis disease collected over a 12-year period (1993–2004). Standard IS*6110* DNA fingerprinting was performed to classify the strains into strain families (>65% similarity) as previously described [20,21]. IS*6110*-RFLP patterns with 100% similarity were considered a cluster and a unique strain has no identical patterns found in the community of the study period. The strains selected were from 4 of the 5 most dominant strain families circulating in community (LAM3; Beijing; Haarlem and S-family) [22,23]. The X-family was excluded since no unique RFLP patterns were present in the community. Isolates were selected from patients who completed treatment or were classified as cured. Patients who transferred out or died were not considered. Furthermore, isolates were selected from the 1st 5 years of the study period, to have a high confidence for the ability to transmit in the study community.

All strains were grown in 7H9 broth containing 0.05% Tween 80, OADC, and glycerol. When cultures reached an OD_600_ reading of 0.600–1.00 they were bottled, frozen, and then titered. Thirteen isolates underwent analysis in this study; these are listed in Table 1. No differences were seen in the relative speed of the cultures to grow in vitro.

**Table 1:** Strains used in this study.

Isolates	Family	Cluster	Designation
509	S	Unique	NOT
923	Haarlem	Unique	NOT
1115	LAM	Unique	NOT
1125	Beijing	Unique	NOT
2252	S	Unique	NOT
385	Haarlem	Unique	NOT
433	S	10	MT
524	S	16	MT
547	LAM	20	MT
3147	Haarlem	18	MT
138	Haarlem	15	MT
1009	Beijing	76	HT
708	LAM	“high”	HT
212	Beijing	155	VHT
3417	Beijing	150	VHT

NOT; No observed transmission.

MT; moderate transmission strain.

HT; high transmission strain.

VHT; very high transmission strain.

A Madison chamber aerosol generation device was used to expose guinea pigs to the different strains of *M. tuberculosis*. This device was calibrated to deliver approximately 10–20 bacilli into the lungs. Thawed aliquots of frozen cultures were diluted in sterile saline to the desired inoculum concentrations. Bacterial loads in target organs were determined by plating serial dilutions of individual whole organ homogenates on nutrient 7H11 agar. CFU were counted after incubation for 3 weeks at 37 °C in humidified air.

### 2.3 RT-PCR analysis

Expression of mRNA encoding the cytokines IFNγ, IL-12p40, IFNα and β, TGFβ, IL-17, IL-10, and Foxp3 was quantified using real-time reverse transcription-polymerase chain reaction analysis on day-30. One lobe from each guinea pig (n = 5) lung was added to 1 ml of TRIzol RNA reagent (Invitrogen), homogenized, and frozen immediately. Total RNA was extracted according to the manufacturer's protocol. RNA samples from each group and each time point were reverse transcribed using the Reverse Transcriptase Enzyme (M-MLV RT- Invitrogen). Four microliter samples of cDNA were then amplified using the iQ SYBR Green Supermix (Bio-Rad) following the manufacturer's protocol on the iQ5 iCycler amplification detection system (Bio-Rad). A negative control using ultra pure molecular biology grade water as the template and a non-template control (NTC) were ran to confirm that the signals were derived from RNA and not due to contaminating genomic DNA. In order to ensure that this was correctly amplified, and was not the presence of primer-dimer or non-specific secondary products, a Melt Curve was performed for each run. Fold induction of mRNA was determined by analyzing cycle threshold (C_T_) values normalized for HPRT (C_T_) expression. The primer sequences for these markers were previously published [14,24,25].

### 2.4 Histologic analysis

The lung lobes from each guinea pig were fixed with 4% paraformaldehyde in phosphate buffered saline. Sections from these tissues were stained using haematoxylin and eosin as previously reported [[26], [27], [28]].

### 2.5 Flow cytometric analysis

To prepare single cell suspensions, the lungs were perfused with 20.0 ml of a solution containing PBS and heparin (50 U/ml; Sigma-Aldrich, St. Louis, MO) through the pulmonary artery and the caudal lobe aseptically removed from the pulmonary cavity, placed in media and dissected. The dissected lung tissue was incubated with complete DMEM (cDMEM media) containing collagenase XI (0.7 mg/ml; Sigma-Aldrich) and type IV bovine pancreatic DNase (30 μg/ml; Sigma-Aldrich) for 30 min at 37 °C. The digested lungs were further disrupted by gently pushing the tissue twice through a cell strainer (BD Biosciences, Lincoln Park, NJ). Red blood cells were lysed with ACK buffer, washed and resuspended in cDMEM. Total cell numbers were determined by flow cytometry using BD™ Liquid Counting Beads, as described by the manufacturer (BD PharMingen, San Jose, CA USA 95131).

Single cell were prepared as previously described [26,27]. The cell suspensions from each individual guinea pig were incubated first with antibodies to CD4, CD8, CD45, MIL4, and B cells at 4 °C for 30 min in the dark and after washing the cells with PBS containing 0.1% sodium azide (Sigma-Aldrich). Propidium iodide was used to discern dead cells from live cells, and an FcγR blocking antibody used [10min on ice] to prevent interference. Data acquisition and analysis were done using a LSR-II flow cytometer (BD Biosciences, Mountain View, CA) and CellQuest software (BD Biosciences, San Jose, CA). Compensation of the spectral overlap for each fluorochrome was done as previously described [29]. Analyses were performed following acquisition of at least 100,000 total events.

### 2.6 Whole genomic expression analysis

Total RNA was purified by digesting contaminating DNA with DNAse followed by isolation of RNA using a Qiagen RNeasy minikit. Total nucleic acids were suspended in nuclease free water to a final volume of 350 μl. An equal volume of 70% ethanol was added to the solution and transferred to an RNeasy column and centrifuged for 1 min at 8000 rpm. The bound material was washed with 350 μl of RW1 wash buffer was added and DNase (Qiagen) treated for 15 min at room temperature. Following DNA digestion, the bound total RNA was washed with 350 μl of RW1 wash buffer, and then washed twice in 500 μl of RPE buffer at 8000 rpm and 13000 rpm respectively. Dry spin was done for 1 min at 13000 rpm. RNA was eluted with nuclease free water.

A customized guinea pig 8 × 60 K (AMADID: 040961) microarray was designed using Genotypic Right Design Technology, and was based on up to date sequences currently available at the NCBI & Ensembl database, thus allowing the design of a unique custom guinea pig microarray. The 60-mer oligonucleotide probes designed were specific to guinea pig genes and EST sequences. Probes were distributed among 6759 genes and 33825 ESTs in the microarray. The final microarray designed consisted of 62976 genomic features including replicated probes for the 8 × 60k Agilent array format.

The concentration and purity of the extracted RNA was evaluated using a Nanodrop Spectrophotometer (Thermo Scientific). The integrity of the extracted RNA was analyzed using a 2100 Bioanalyzer (Agilent). RNA quality was assessed based on 260/280 values, rRNA 28S/18S ratios, and RNA integrity number. The samples were then labeled using Agilent Quick Amp Kit (Part number: 5190-0442) and then 500 ng of total RNA was reverse transcribed using oligodT primer tagged to the T7 promoter sequence. cDNA thus obtained was converted to double stranded cDNA in the same reaction. Then, the cDNA was converted to cRNA in an in-vitro transcription step using T7 RNA polymerase enzyme, and then Cy3 dye was added into the reaction mix. A single color Quick-Amp Labeling kit [Agilent] was used for the cDNA preparation following the manufacturer's guidelines. During cRNA synthesis the Cy3 dye was incorporated into the newly synthesized strands. cRNA obtained was further purified using a Qiagen RNeasy column. The concentration and amount of dye incorporated was determined using Nanodrop. Samples that passed quality control measures for specific activity and yield were hybridized on the customized guinea pig 8 × 60k array designed in-house using an Agilent Gene Expression Hybridization kit at 65 °C for 16 h. Hybridized slides were then washed using Agilent Gene Expression wash buffers. The microarray slides were then scanned on an Agilent G2600D scanner. Raw data extraction from the scanned images was done using Agilent Feature Extraction software (Version 11.5).

Feature extracted data was analyzed using Agilent GeneSpring GX software. Normalization of data was done using the 75th percentile shift method (percentile shift normalization is a global normalization, where the locations of all the spot intensities in an array are adjusted); this process performs the baseline transformation before calculating the fold change. Significant differentially regulated genes that were either up or down regulated showing one fold or above changes in expression with significant P-values (<0.05) within the group of samples were identified. Statistical P-values were calculated using Student's *t*-test method and the Hochberg method. Differentially regulated genes were clustered using hierarchical clustering based on the Pearson coefficient correlation algorithm to identify significant gene expression patterns. Differentially regulated genes were classified into different functions based on their Gene Ontology functional category.

### 2.7 Statistical analysis

Differences in survival between groups of experimental animals was performed by Kaplan Meier analysis.

## 3 Results

### 3.1 Growth of clinical isolates in the low dose aerosol guinea pig model

**Fig. 1:**
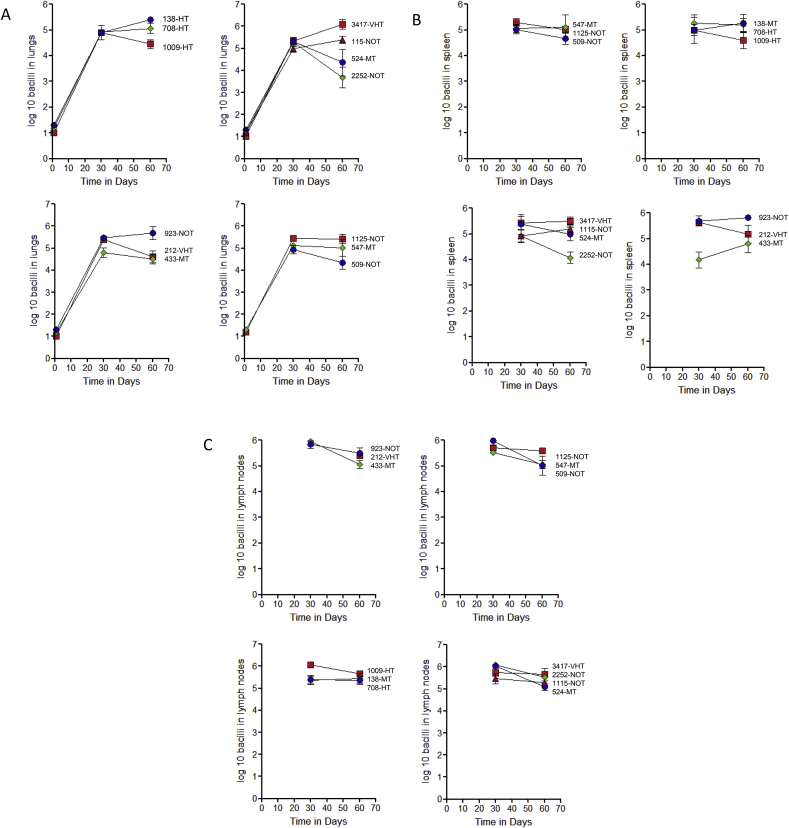
Growth of unique [NOT; no observed transmission], moderate transmission [MT], high transmission [HT] and very high transmission [VHT] clinical isolates of *M. tuberculosis* in the lungs of guinea pigs after deposition of approximately 15–20 bacilli by aerosol exposure. Data is shown as mean values from five animals per time point ±SEM, in the lungs [Fig. 1A], spleen [Fig. 1B], and draining lymph nodes [Fig. 1C].

We systematically analyzed the growth characteristics of each isolate in the panel by exposing guinea pigs to 15–20 bacilli using a Madison chamber. The bacterial load was determined in the lungs, spleen, and draining mediastinal lymph nodes 30 and 60-days later and plotted as log-10 mean values. As shown in Fig. 1A all the isolates tested grew to a bacterial load of ∼10^5^ by 30-days, and thereafter either remained at a similar level or underwent some degree of clearance. Very similar numbers were seen disseminating to the spleen (Fig. 1B) or the draining lymph nodes (Fig. 1C). Based on these results it was evident that NOT strains, moderately transmitted strains [MT], or HT strains could not be distinguished from each other based on growth alone.

### 3.2 Pathology of the lung in animals infected with HT and LT isolates

**Fig. 2:**
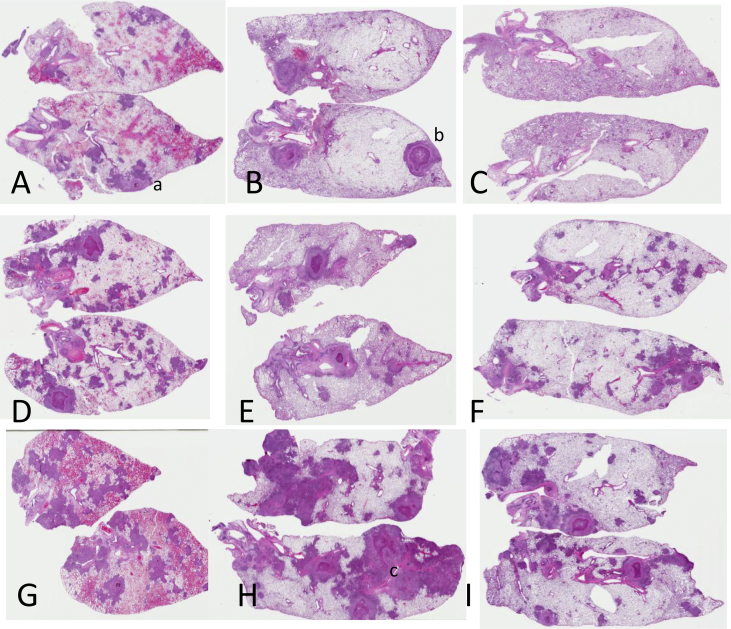
Representative whole lung scans from infected guinea pigs harvested on day-60 of the infection. Hematoxylin and eosin staining. The top row [A-C] are high transmission strains 708, 1009, and 212. The middle line [D-F] are moderately transmitted strains 138, 433, and 547. The bottom row [G-I] are unique strains 923, 1125, and 1115, in which no transmission has been observed. Features in A-C consist of scattered granulomas, some on the pleural surface [“a”] with others showing obvious central necrosis [“b”]. In D-F there are substantially more lesions, and in G-I some of these are severe and there is obvious granulomatous consolidation [“c”].

Each group of infected animals was analyzed for lung histopathology. The complete results of these studies are shown in Supplementary Fig. 1. Some representative results are shown in Fig. 2, and are arbitrarily broken down into day-60 lung histology seen in HT isolates [A-C], isolates showing relatively modest patterns of transmission [D-F], and isolates presenting as unique [G-I].

We had predicted that HT strains would cause the most lung damage, thus allowing them to escape the lungs, but in fact the results suggested the reverse. In most cases lung pathology elicited by HT strains was relatively modest, with only a few necrotic lesions visible on day-60. Other isolates, associated with moderate transmission patterns, seemed to cause more damage, with multiple lesions throughout the lungs, some of which were in the process of necrotizing. Three further isolates, all unique with no known transmission, caused moderate to severe lung damage characterized by multiple necrotic lesions.

### 3.3 RT-PCR analysis of key host molecules

**Fig. 3:**
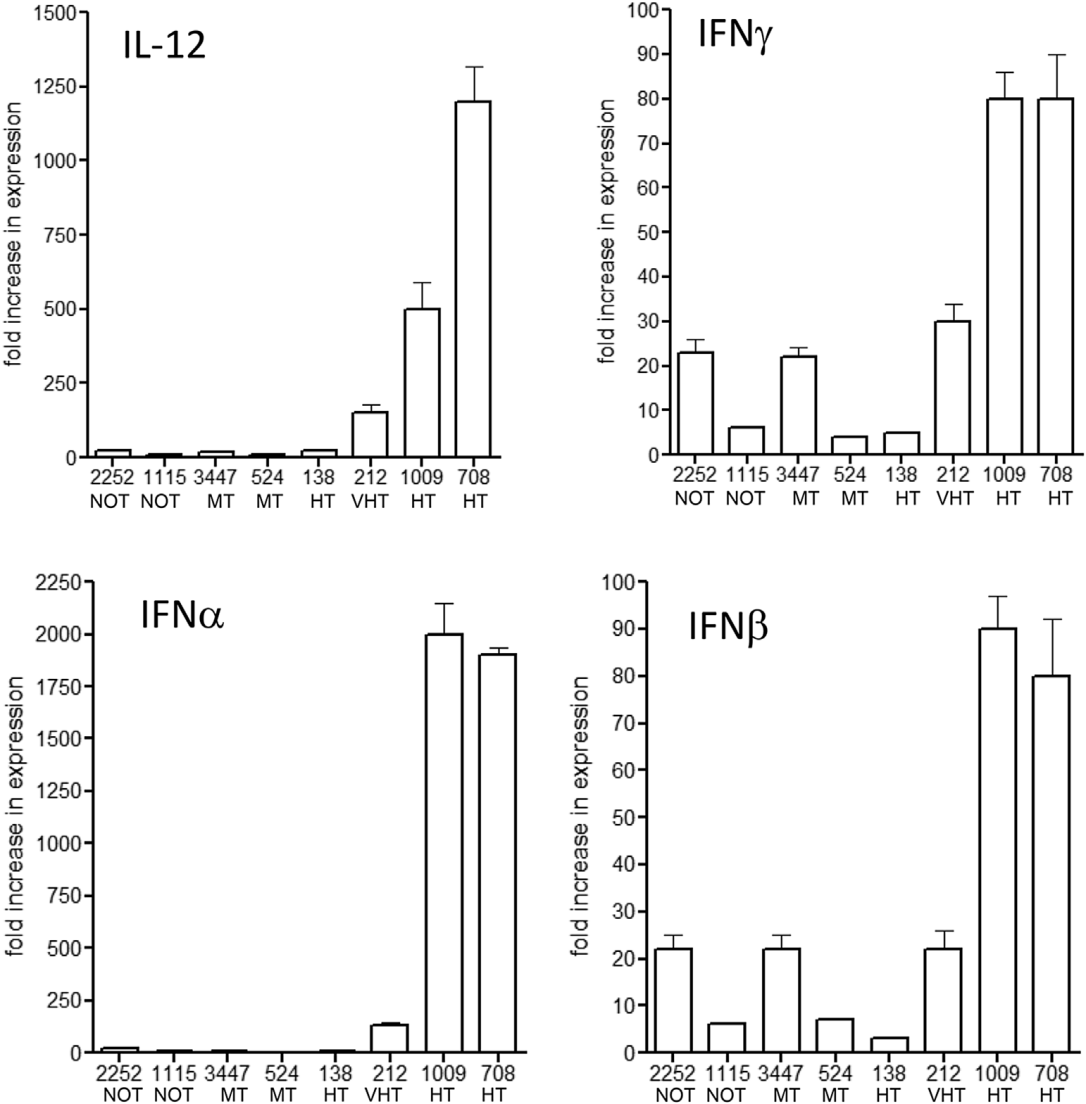
RT-PCR signals for key TH1-associated responses in the lungs of guinea pigs infected with a panel of clinical strains of *M. tuberculosis*. Data show as mean of three measurements ±SEM.

**Fig. 4:**
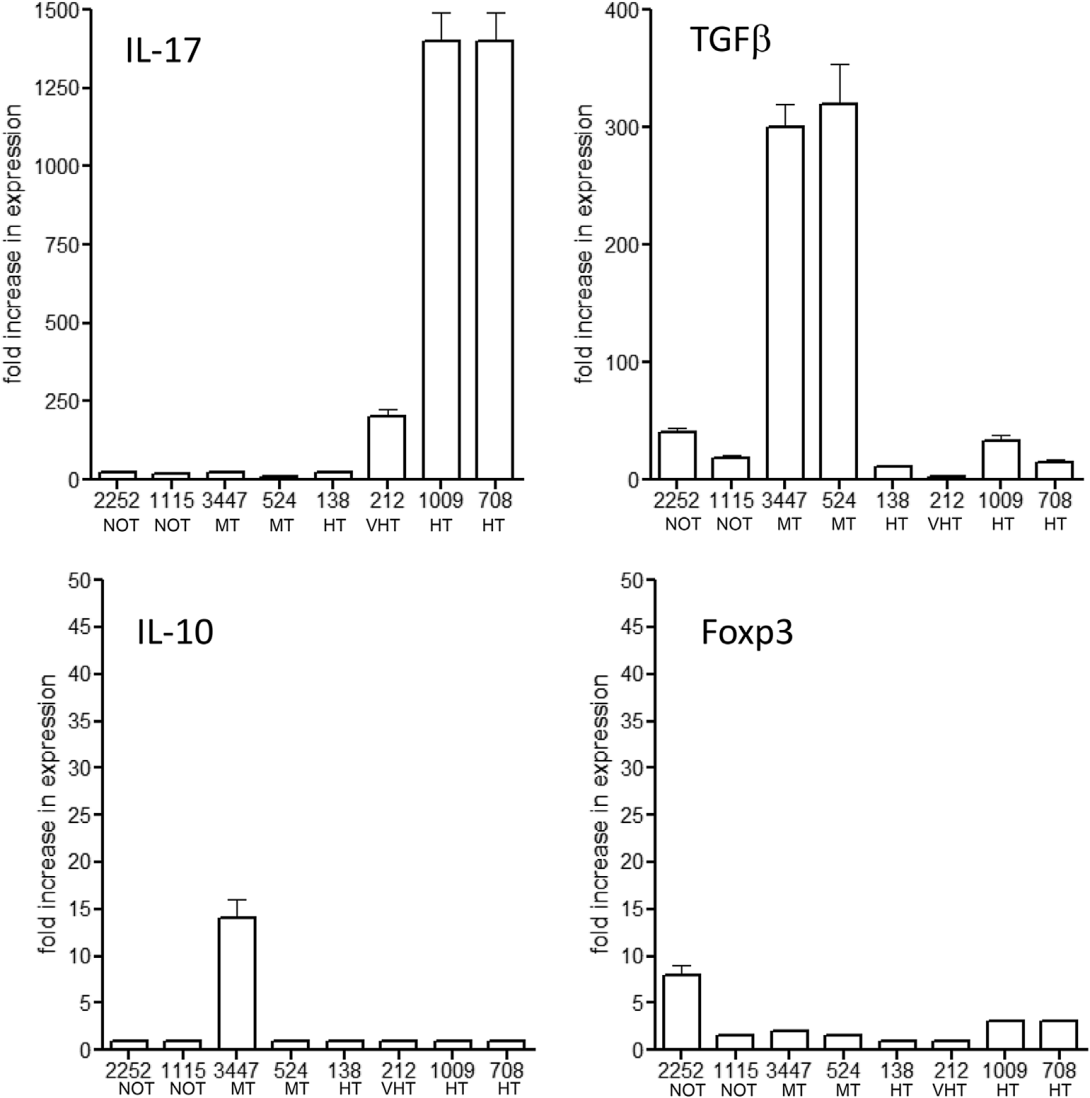
RT-PCR signals for key regulatory responses in the lungs of guinea pigs infected with a panel of clinical strains of *M. tuberculosis*. Data show as mean of three measurements ±SEM.

In several cases we isolated lung lobes and performed RT-PCR analysis. As shown in Fig. 3, the highest signals for the TH1 cytokines IL-12 and IFNγ were seen in response to some, but not all, HT isolates, and this was also the case for the type-I interferons. In the case of molecules associated with immunoregulation, much higher signals for IL-17 were seen in response to HT strains (Fig. 4). In general, signals for TGFβ were very low, except in the case of two moderately transmitted strains. Virtually no signals were generated by any of the isolates for the regulatory T cell associated markers IL-10 and Foxp3.

### 3.4 Flow cytometric analysis of the host response in selected HT and LT isolates

**Fig. 5:**
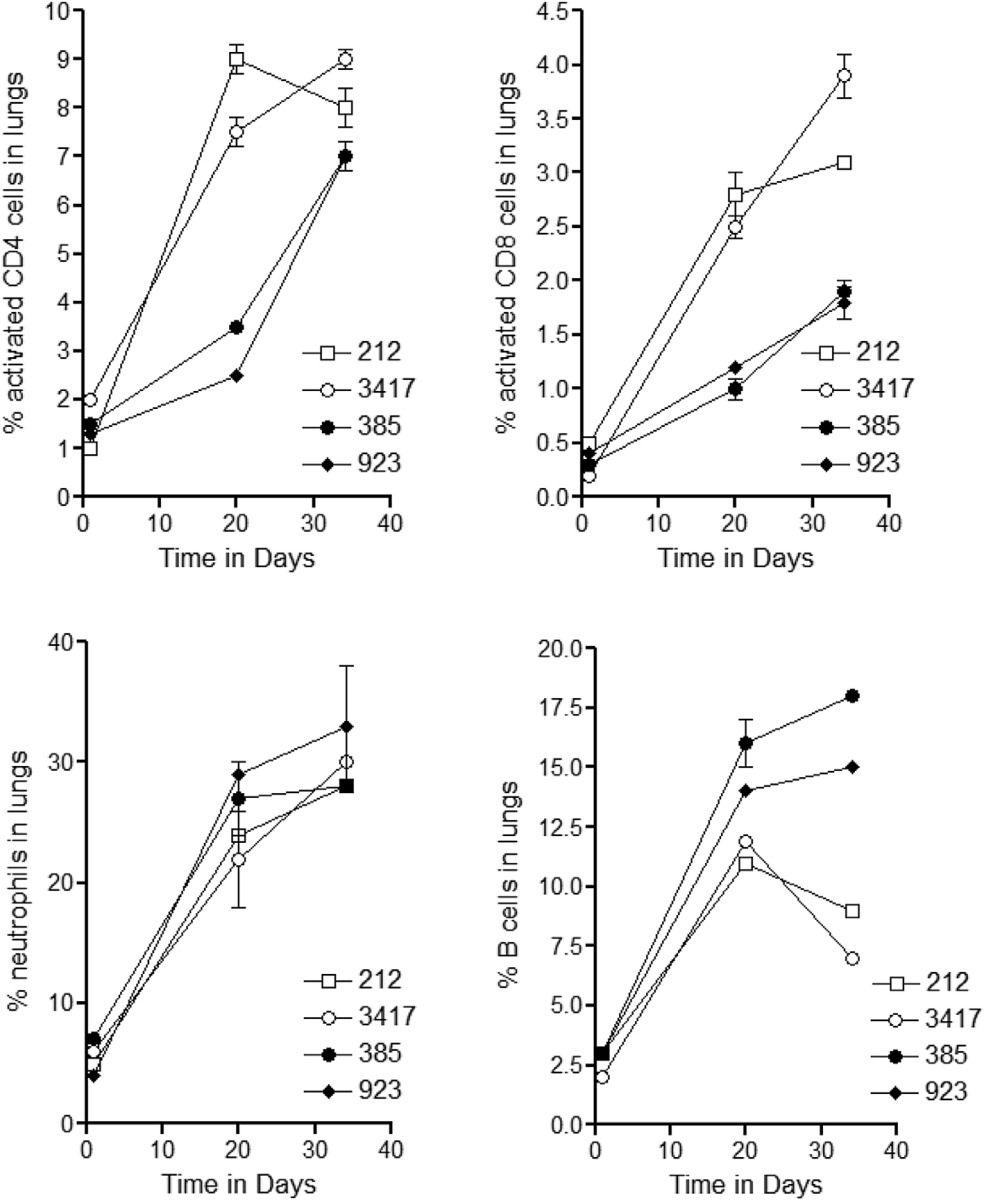
Flow cytometric analysis of the leukocyte response in the lungs during the course of infection with very high transmission strains [212, 3417] or unique strains [385, 923]. Data shown as the mean of 5 measurements ±SEM.

Two HT and two NOT isolates were selected arbitrarily and used for flow cytometric and genomic studies. Clear differences were observed (Fig. 5) between the two groups in terms of influx of activated [CD45^hi^] CD4 and CD8 T cells into the lungs over the first 34-days, with evidence for a substantially accelerated response to the two HT isolates. In terms of B cell influx, a known component of the tuberculosis granuloma [30], relatively similar numbers were seen in response to all four isolates by day-20; these declined in the HT animals while continuing to rise in the NOT group. Because it is still unclear what the mechanism is that attracts B cells into the lungs we cannot draw any conclusions here, although we should point out that the drop in B cell numbers in the HT groups did not appear to affect the overall patterns of granulomatous inflammation. Finally, in all four cases, neutrophil influx was similar.

### 3.5 Genetic response analysis to selected HT and LT isolates

**Fig. 6:**
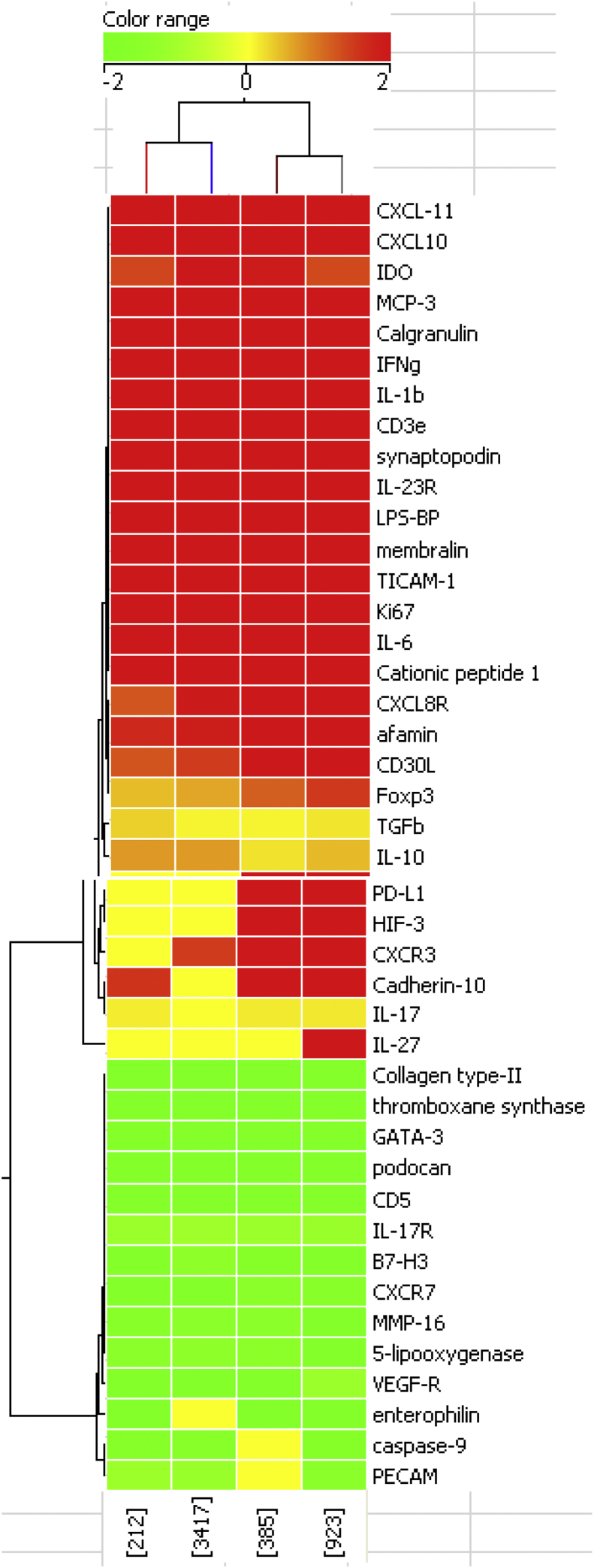
Whole genomic analysis of the response in guinea pigs to two very high transmission strains [left panels] or two unique strains [right panels]. Data is shown as a heat map for major molecules of interest. Note the very high expression of PD-L1 and HIF-3 in the unique strains, absent in the animals infected with the high transmission strains.

Lungs were harvested from the four studies above on day-30 and subjected to whole genomic analysis, as previously described [31].> What was immediately noticeable was that the number of genes turned on or off by the NOT isolates was far higher than that of the HT isolates (Table 2). Within these, multiple genes of interest showed increased expression (Table 3) while others were clearly down-regulated (Table 4). The most active of these are shown as a heat-map in Fig. 6.

**Table 2:** Total genes showing changes in expression.

Isolate		#increased expression	#decreased expression
212	HT	1270	1809
3417	HT	1530	1713
385	LT/U	3216	3097
923	LT/U	2938	3847

**Table 3:** Genes of interest with highest fold-increase in expression.

Gene	923	385	3417	212
CXCL-11	4.26	5.02	5.86	4.30
Calgranulin	5.09	4.73	4.63	4.29
IL-6	3.76	3.24	4.81	4.30
Cationic peptide-1	4.76	2.99	3.50	2.95
MCP-1	2.27	2.91	4.22	2.42
IFNγ	4.11	3.98	4.13	3.41
LPS-BP	3.82	3.40	4.03	2.68
Membralin	3.80	4.04	3.24	3.70
PD-L1	3.43	3.38	0	0
CD3ε	3.38	2.70	2.96	2.92
CXCLR-8	2.91	2.29	2.4	2.47
Synaptopodin	2.77	2.29	2.49	2.40
IL-1β	2.69	2.41	2.79	2.30
Afamin	2.67	2.68	1.87	1.70
HIF-3	2.56	2.14	0	0
TICAM-1	2.54	2.80	2.48	2.72
IL-23R	2.51	2.22	2.16	2.38
CD30L	2.50	2.47	1.47	1.25
CXCL-10	2.40	3.08	3.70	2.56
Cadherin-10	2.30	2.59	0	1.56
CXCL-8R	2.24	2.15	1.50	0
Ki67	2.07	2.77	2.48	2.27
IL-27	2.03	0	0	0
CXCR3	2.02	1.97	1.47	0
IDO	1.35	1.93	2.19	1.38
TGFβ	0.20	0.10	0.10	0.34
IL-17	0.18	0.17	0.01	0.15
IL-10	0.48	0.21	0.70	0.71
Foxp3	1.50	1.16	0.62	0.47

**Table 4:** Genes of interest with fold-decrease in expression.

Gene	923	385	3417	212
Collagen type-II	4.11	4.66	3.95	3.95
CD5	2.87	2.94	2.79	3.21
enterophilin	3.51	3.63	0	2.76
GATA-3	2.31	2.47	2.06	1.84
caspase-9	1.76	0	1.67	1.72
PECAM	1.55	0	1.17	1.11
MMP-16	2.18	1.54	1.54	1.64
thromboxane synthase	2.32	2.33	2.15	2.01
VEGF-R	1.14	2.34	2.31	2.03
CXCR7	1.66	1.52	1.88	1.86
IL-17R	1.16	1.13	1.08	1.12
B7-H3	1.90	1.79	1.42	1.85
5-lipooxygenase	1.94	1.48	1.41	1.55
podocan	2.33	2.72	2.57	2.42

As part of this process we looked for gene signatures that could clearly delineate between HT and NOT strains. However, only two seemed to have this property – PD-L1 and HIF-3 – which were strongly upregulated in the NOT group but remained unchanged in the animals infected with the HT isolates.

## 4 Discussion

The objective of this study was to determine if differences could be observed in the host response in the guinea pig to infection with isolates of *M. tuberculosis* that differ in their known transmission histories. To determine this we studied a panel of multiple isolates that showed a large range in transmission across the Western Cape region, and then randomly selected four of these for more comprehensive investigation, including whole genomic analysis.

The isolates used in these analyses originated from a community study centered in Cape Town, South Africa. Strains were selected based on findings from a longitudinal study over 12 years, which described the strain diversity [21], transmission events in the community [32], and outbreaks [33]. We selected strains from large clusters (>15 patients per cluster) that were highly transmissible, from a collection of the most common strain families in the local community and the Western Cape Province as a whole. Together with these clusters, we also selected strains that were unique in the community, defined as no evidence of transmission over the study period, from the same strain families as the transmitted strains. Included in this selection were representatives from the Beijing, LAM3, S family, and Haarlem strain families. The Beijing, LAM3, S-family, together with the X-family are also strains that are predominantly represented in the current drug-resistant tuberculosis epidemic in the Western Cape Province [22,23,34].

The first obvious conclusion from our studies is that transmission patterns are not linked to the intrinsic virulence of the individual organism, in that all the strains we tested grew well after low dose aerosol exposure of guinea pigs. That is not to say that there might not be differences in virulence, just that the guinea pig model is not able to distinguish this. However, despite the similar CFU levels seen for all the isolates, there appeared to be some observable differences in the lung pathology between the various groups of strains. In general, in most cases the lung pathology appeared to be much more severe in animals infected with NOT strains, and mostly only mild to moderate in the strains with higher transmission levels. This result is hard to explain – one would expect the reverse. Given the data we have, we are forced to hypothesize that the substantial lung damage and multiple large necrotic granulomatous lesions induced by the NOT isolates has as its basis a florid inflammatory response which is coupled with a slower and smaller expression of acquired immunity. This is in contrast to our results with the HT strains, which seem to suggest that a much faster expression of T cell mediated immunity resulted in rapid containment of these strains with minimal granulomatous involvement.

Although these strains were chosen arbitrarily, both HT strains were members of the Beijing family, and the NOT strains were Haarlem strains. Beijing strains are of course known to highly transmitted worldwide [17], but the incidence of Haarlem strains is also considerable. Given the small sample sizes here one cannot conclude anything, but if our data represents a trend, then Beijing strains are of high virulence, high immunogenicity, but low pathogenicity, whereas Haarlem strains are high virulence, low immunogenicity, and of high pathogenicity.

There were some clear differences in our results using the guinea pig model with earlier studies in mice. In this regard, it has been known for some time that, in contrast to the laboratory strains of *M. tuberculosis*, clinical strains can exhibit a wide range of virulence and pathogenicity when tested in mice. Even before strains were typed into families it was found that many drug-resistant strains were virulent in the mouse model when compared to the Erdman strain [35]. More recently, these differences were found using a high dose intra-tracheal infection model in susceptible BALB/C mice. Infections were rapidly fatal in animals infected with Beijing strains, whereas this event was much slower in mice infected with Haarlem or Somali isolates [8], although despite this the actual increase in the bacterial load in the lungs was relatively modest [0.5–1.5-log]. Improved bacterial growth was then observed in a further study [36] and here high virulence correlated with worsened lung pathology. Similar findings were found in a later study [9] which linked virulence to transmission, whereas in contrast avirulent strains were controlled by strong TH1 immunity. More recently [6] an interesting study showed that mice infected with highly transmitted Beijing isolates could co-infect cage mates, although whether this was due to regurgitation/exhalation of the intra-tracheal inoculum bolus is unclear [since mice do not cough]. These strains were rapidly fatal in these mice despite very modest increases in lung CFU, which might be reflecting the severe lung pathology the high transmission strains induced.

We have recently tried to reconcile the three factors that we feel control the outcome of initial infection with clinical strains [37] -- these are intrinsic isolate virulence, isolate immunogenicity, and isolate fitness. All members of our panel grew equally well and appeared equally virulent. Our collective studies to date lead us to think that many of the Western Cape strains are of relatively low fitness – at least in the context of their capacity to grow in the face of acquired immunity -- mainly based on the fact [7,15] that BCG vaccination in mice and guinea pigs is strongly inhibitive [at least for a while]. Our results here indicate that HT isolates are highly immunogenic, given the rapid expansion and activation of lung T cells observed here, whereas NOT isolates are far less so.

One can speculate that if an isolate is of low fitness and low immunogenicity, but can induce inflammation, the T cell response would be expected to be slower and smaller and the inflammatory response in the lungs [primarily neutrophil-mediated] would favor the development of large and necrotic granulomas, from which these isolates do not tend to be able to escape [30]. Then, consider in contrast an isolate that is also relatively low fitness, but highly immunogenic. Here, immunity would be expressed quickly and granulomatous inflammation would be much reduced [in this regard, we previously demonstrated that protection can be expressed even when granulomatous inflammation is absent [38]]. Under these conditions the infection would be initially contained, but bacilli surviving would find themselves in an environment in which escape, and hence transmission, would be far easier. In other words, being immunogenic may confer an advantage in terms of transmission. This at first seems contrary to logic, but it is in fact consistent with the knowledge [39] that clinical isolates conserve immunodominant epitopes regardless of strain family. A study of the degree of epitope conservation between HT and NOT strains might answer this question further.

Along these same lines, a further factor may be the intrinsic inflammatory nature of the bacillus itself. The NOT isolates in general created moderate to severe inflammatory lesions, whereas the HT isolates mostly did the reverse. This suggests that preventing an inflammatory focus at the site of infection may be an advantage to the bacterium, preventing the influx of cells that would otherwise prevent escape into the tidal air and subsequent transmission.

Given our previous experience [1,2] with “US outbreak” strains, we predicted that African strains in general, and HT strains in particular, would be expected to turn on regulatory immunity, which we would detect by increased signals for IL-10 and [particularly] Foxp3 by PCR. This was not the case at all, and these observations were confirmed by our whole genomic sequencing analysis. This observation further supports our growing feeling that strains from the Western Cape are relatively low in “fitness”. This adds a further variable, namely the environment in which the isolates become spread. For instance, isolates obtained [40] in the Bay Area [California] are transmitted amongst a [mostly Chinese] population in which nutrition is good and HIV rates low, and so these isolates seem to have evolved the capacity to generate regulatory T cell activity – which we now know can directly target acquired protective immunity [41] -- in order to survive and persist. In the Western Cape there is malnutrition and high rates of HIV, so regulatory T cell responses are not a factor and the [admittedly limited] strains we have tested so far do not induce them to any extent.

In the studies reported here, we had sufficient resources to conduct whole genomic sequencing analysis to four selected strains. The first surprise was that the total numbers of genes that changed expression was *three times higher* in the response to the NOT strains compared to the HT strains; an observation for which we have no explanation as yet. The heat map signature obtained illustrates the expression of selected genes in the lungs of guinea pigs infected 25-days earlier with HT [212, 3417] or NOT [385, 923] strains. Some changes were expected – strong chemokine responses [CXCL10, CXCL11, MCP-3], IFNγ, IL-6, etc, but others were surprising. IDO has been strongly implicated in immunity to tuberculosis [42,43], but usually in the context of regulatory T cells, surprising because our PCR data showed no significant Foxp3 signal. Gene expression for IL-17 was modest, but the expression of the IL-17-receptor was strongly down-regulated, perhaps indicating a mechanism to dampen inflammation and neutrophil influx, which we have argued [30,44] is the basis of the eventual lung necrosis.

Some genes were strongly down-regulated across all four infections. Collagen type-II, in this context at least, could be being down-regulated to prevent lung repair, which would reduce the influx of host immune cells [as would MMP-16]. Both thromboxane synthase and 5-lipooxygenase play key roles in the production of prostaglandins [45], which can interfere with the expression of cell mediated immunity, and this could explain their down-regulation to prevent this. GATA-3 drives the TH2 response, of course not needed here, as neither is B7-H3, a known check-point molecule. All four strains induced strong induction of LPS-BP, which is curious but might indicate disruption of the upper respiratory tract Gram-negative microbiome. Others, such as membralin and afamin, are barely mentioned in the literature, and not associated to date with tuberculosis infection.

We found two examples where gene expression differed between the two groups – HIF-3, and PD-L1. The HIF factors are the master regulators in tissues exposed to hypoxia, usually occurring as heterodimers. The HIF-1 and HIF-2 molecules are well characterized, but far less is known about HIF-3 [exacerbated by the fact that there appear to be several variants] [46,47]. We previously showed [48] that areas of the infected guinea pig lung granuloma becomes hypoxic [or at least has lowered oxygen tension] and hypoxia has been shown to increase HIF-3 levels in this organ. HIF-3 seems to induce a specific translational signature, and many of the up-regulated genes are thought to be involved in lung development and repair [47]. Given our observation of lung damage in the NOT infected animals, this might explain why these animals upregulate expression of HIF-3, whereas the HT animals did not. In addition, both HIF factors and PD-L1 are promotors of macrophage polarization to the “M1 state” [49], but what is paradoxical here is one would expect the HT group, given their strong TH1 response, to increase PD-L1. Perhaps a better explanation is that the primary role of PD-L1 in this context is to dampen the lung inflammation and damage occurring in the NOT infected animals. Could this be exploited as new biomarkers? It is too early to say, since we would need to demonstrate that this pattern is expressed consistently between HT and NOT infected animals, and moreover, in the blood.

To summarize, based on the limited information we have so far, it is apparent that NOT strains are far more inflammatory that HT strains, switching on a far larger number of genes, some of which almost certainly involved in the observed damage to the lungs and progressive pathology. In contrast, HT strains, while equally virulent, are more immunogenic and develop faster and larger protective T cell responses, while keeping lung damage in the mild to modest range. As discussed, one of course might well think that strains that damage the lungs are more likely to be exhaled and give rise to transmission, and yet the data here paradoxically suggests that in fact the reverse happens. If it is indeed the case that a mild inflammatory response fails to provide sufficient containment of the infection, not only would this be a game changer, but might provide brand new ideas as to how to break the chain of disease transmission.

## Funding

This study was supported by a generous grant from the Bill and Melinda Gates Foundation. The funders had no role in study design, data analysis, decision to publish, or preparation of the manuscript.

## Author contributions

CAS, MHT, and IMO designed the studies. EMS provided the defined clinical strains. CAS, MHT, DS, DJO performed the animal infection studies. CB and RM performed the genetic analyses. MHT, EMS, and IMO wrote the manuscript.

## Competing interests

Genotypic Technology Pvt Ltd is a for-profit company. The other authors have no competing interests.

## Ethical approval

The animal studies reported here were approved by the Animal Care and Usage Committee, and the Biosafety Committee, at Colorado State University.

## Data availability

The entire datasets from the WGS analysis are available upon request to interested parties. Please contact IMO.

## One sentence summary

High and low transmission clinical isolates from the Western Cape region do not differ in terms of virulence in infected guinea pigs, but do differ in terms of host response, pathogenicity, and gene expression profiles.

## Appendix A Supplementary data

**Supplementary Fig. 1**. Lung pathology results. Top eight panels are the two lobes from each of four animals harvested on day-30, and the bottom eight panels are from animals harvested on day-60.

mmc1 mmc1
