# Supplementary material for: Biology of clinical strains of Mycobacterium tuberculosis with varying levels of transmission
Source: Tuberculosis (Edinb). 2018 Mar;109:123–33. doi: 10.1016/j.tube.2018.02.003 (PMC5884417; doi:10.1016/j.tube.2018.02.003)
Supplement: mmc1 [file mmc1.pptx]

## Slide 1
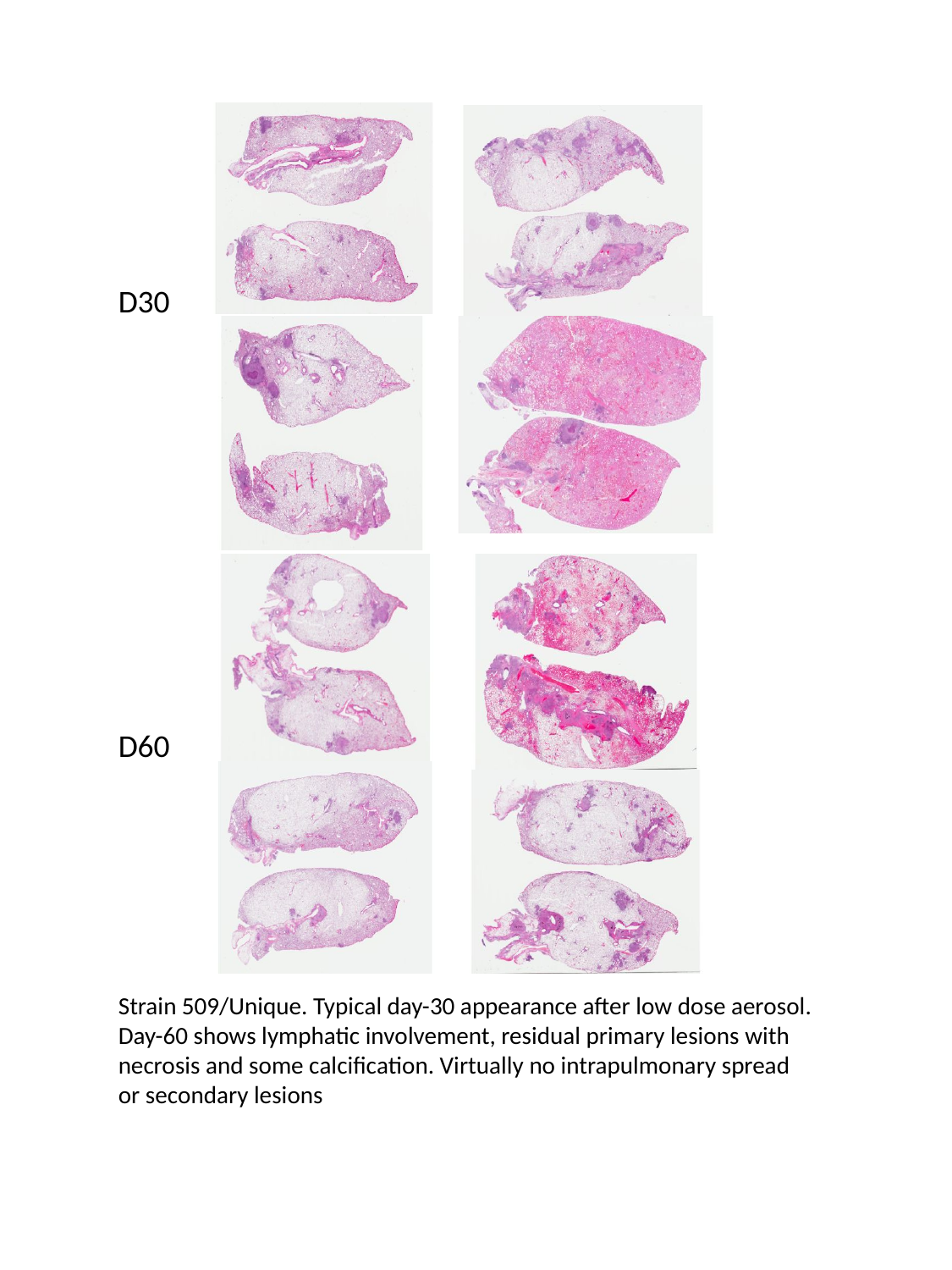

D30
D60
Strain 509/Unique. Typical day-30 appearance after low dose aerosol. Day-60 shows lymphatic involvement, residual primary lesions with necrosis and some calcification. Virtually no intrapulmonary spread or secondary lesions

## Slide 2
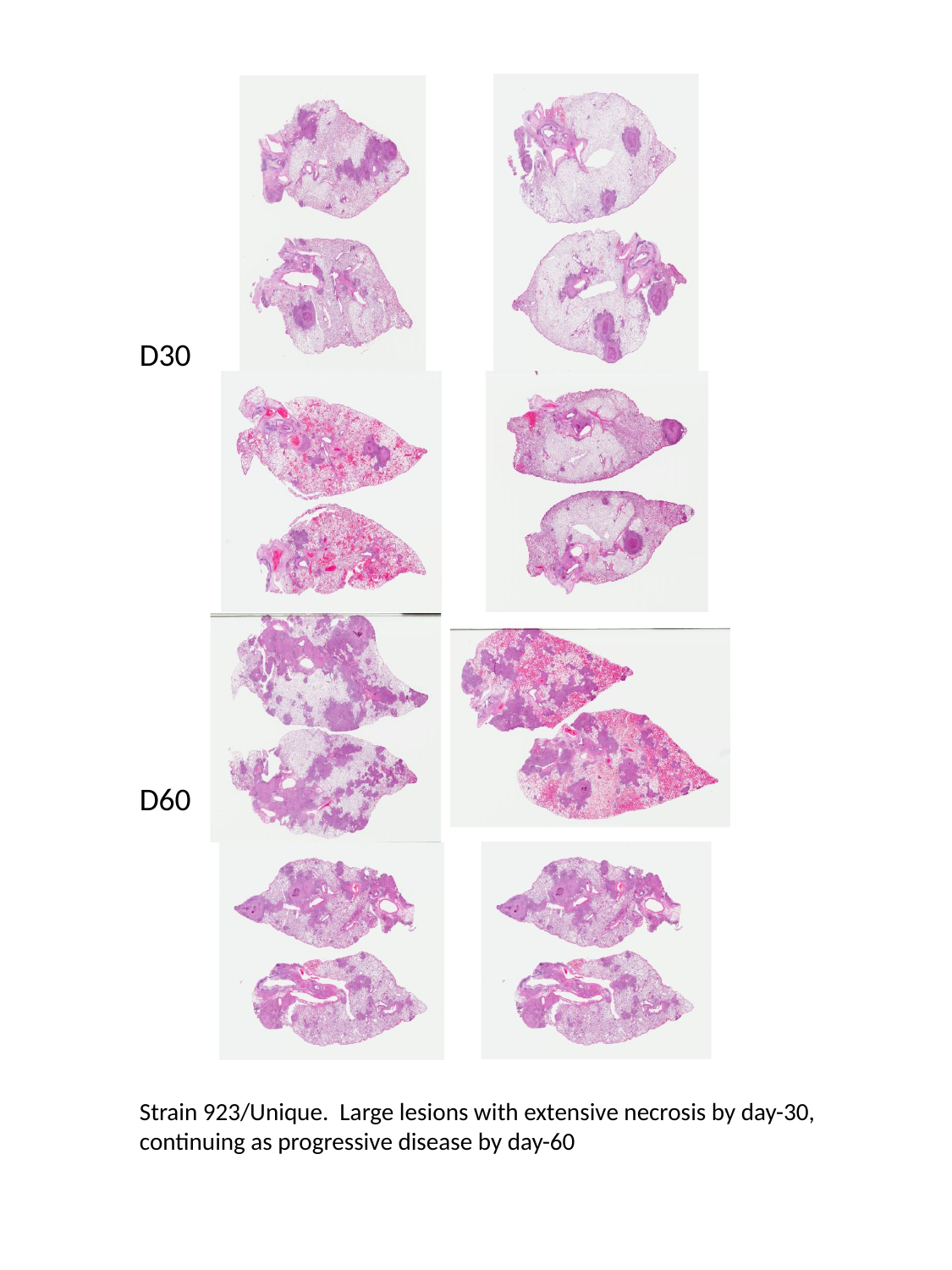

D30
D60
Strain 923/Unique. Large lesions with extensive necrosis by day-30, continuing as progressive disease by day-60

## Slide 3
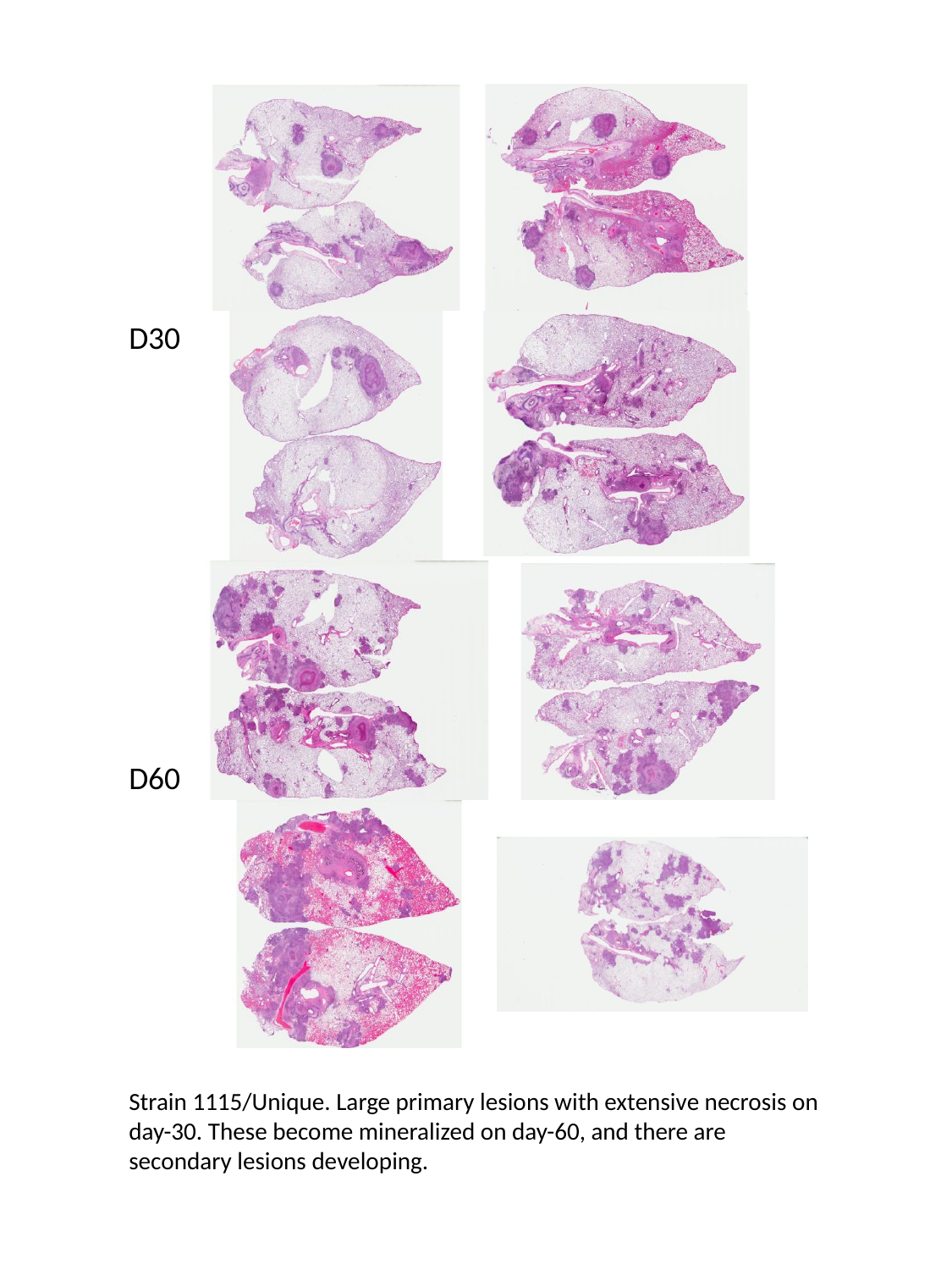

D30
D60
Strain 1115/Unique. Large primary lesions with extensive necrosis on day-30. These become mineralized on day-60, and there are secondary lesions developing.

## Slide 4
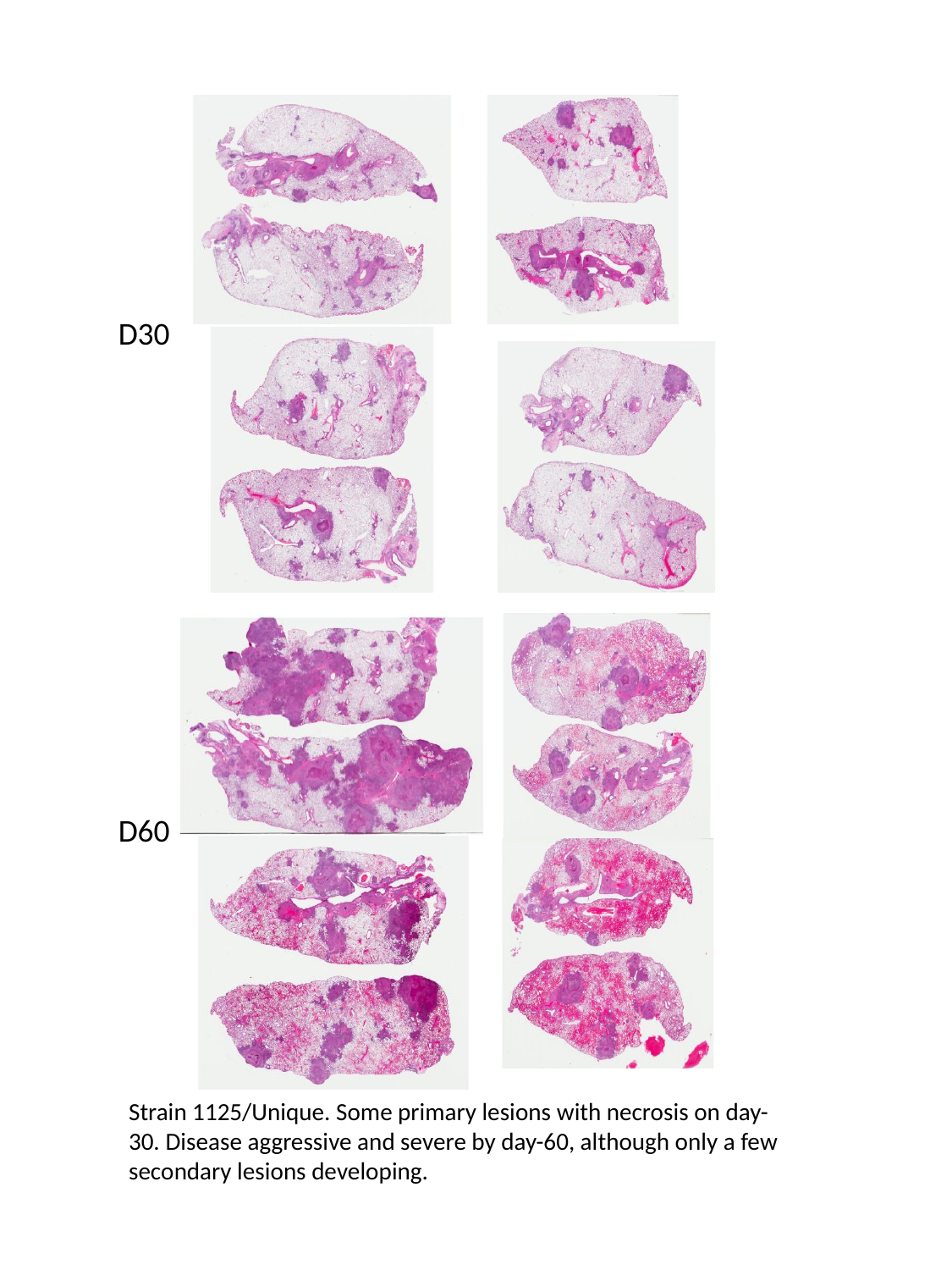

D30
D60
Strain 1125/Unique. Some primary lesions with necrosis on day-30. Disease aggressive and severe by day-60, although only a few secondary lesions developing.

## Slide 5
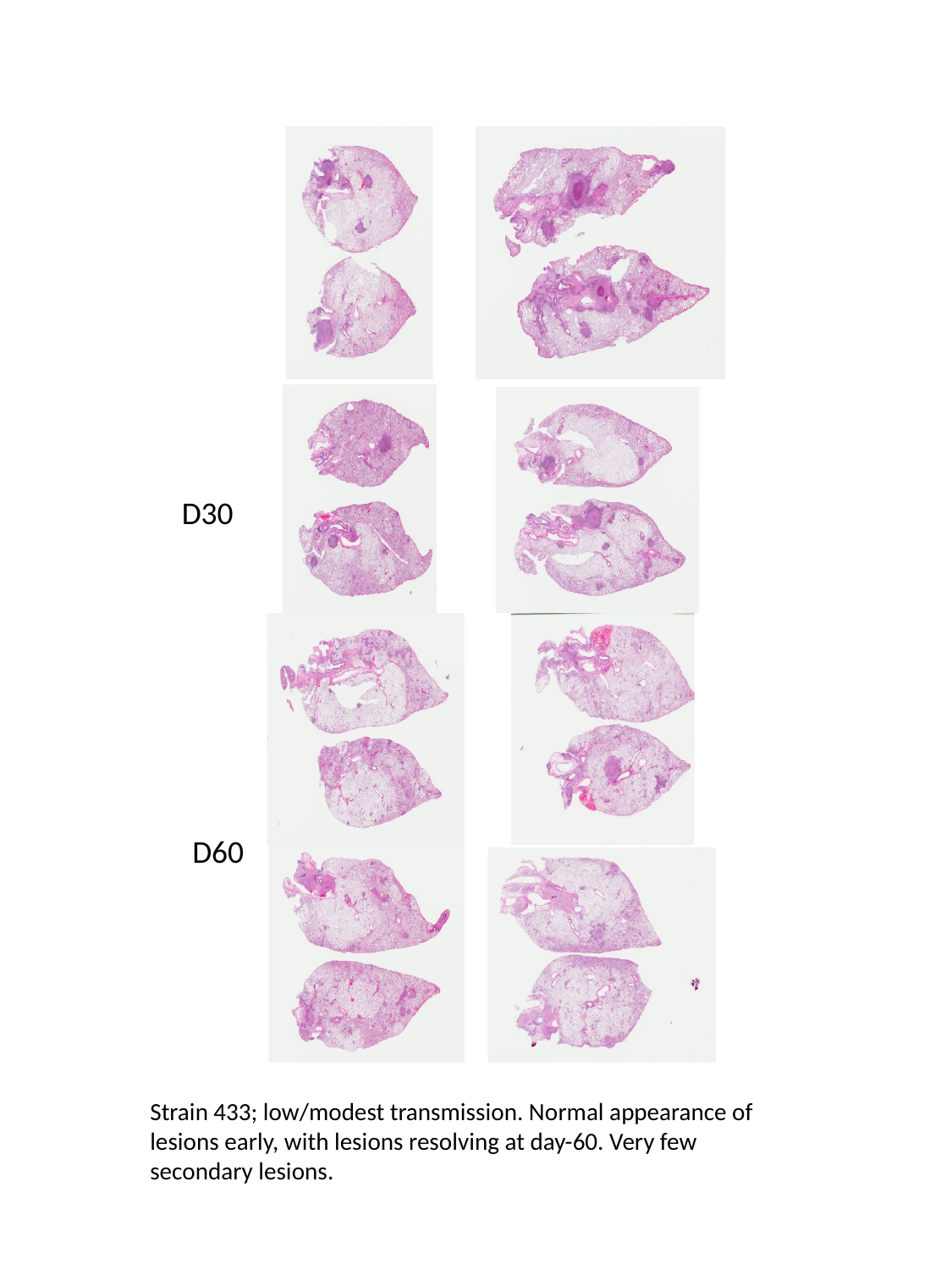

D30
D60
Strain 433; low/modest transmission. Normal appearance of lesions early, with lesions resolving at day-60. Very few secondary lesions.

## Slide 6
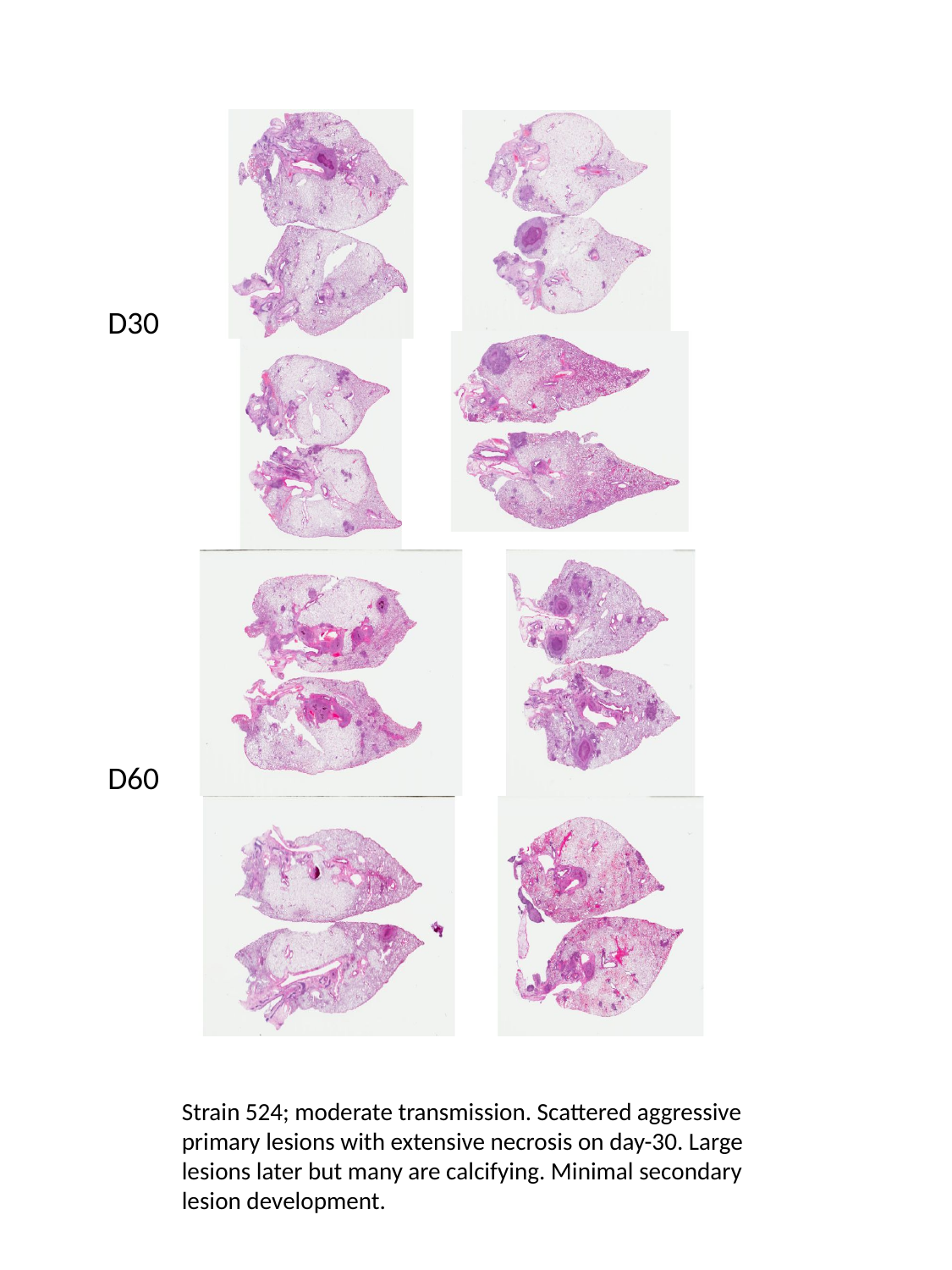

D30
D60
Strain 524; moderate transmission. Scattered aggressive primary lesions with extensive necrosis on day-30. Large lesions later but many are calcifying. Minimal secondary lesion development.

## Slide 7
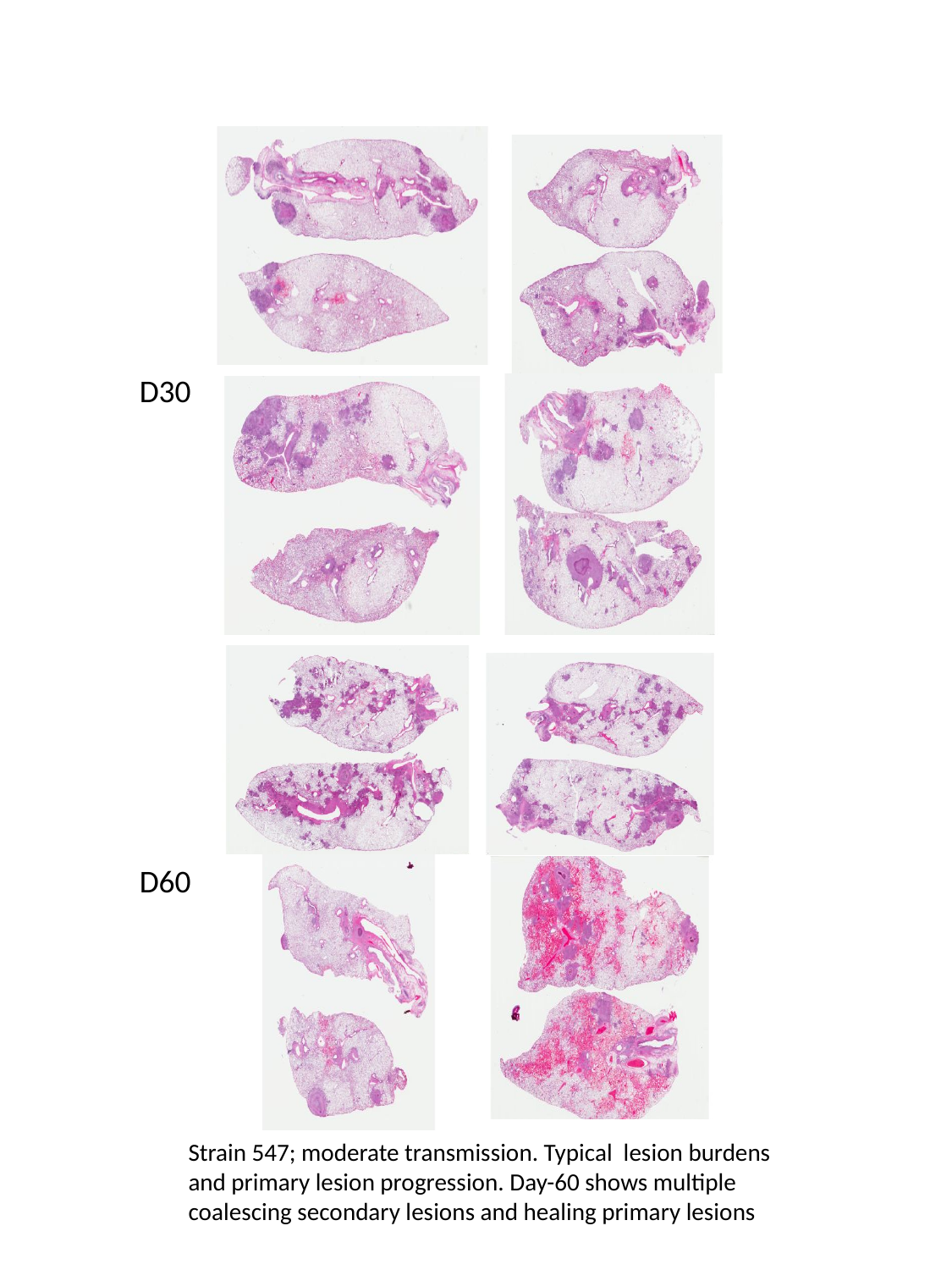

D30
D60
Strain 547; moderate transmission. Typical lesion burdens and primary lesion progression. Day-60 shows multiple coalescing secondary lesions and healing primary lesions

## Slide 8
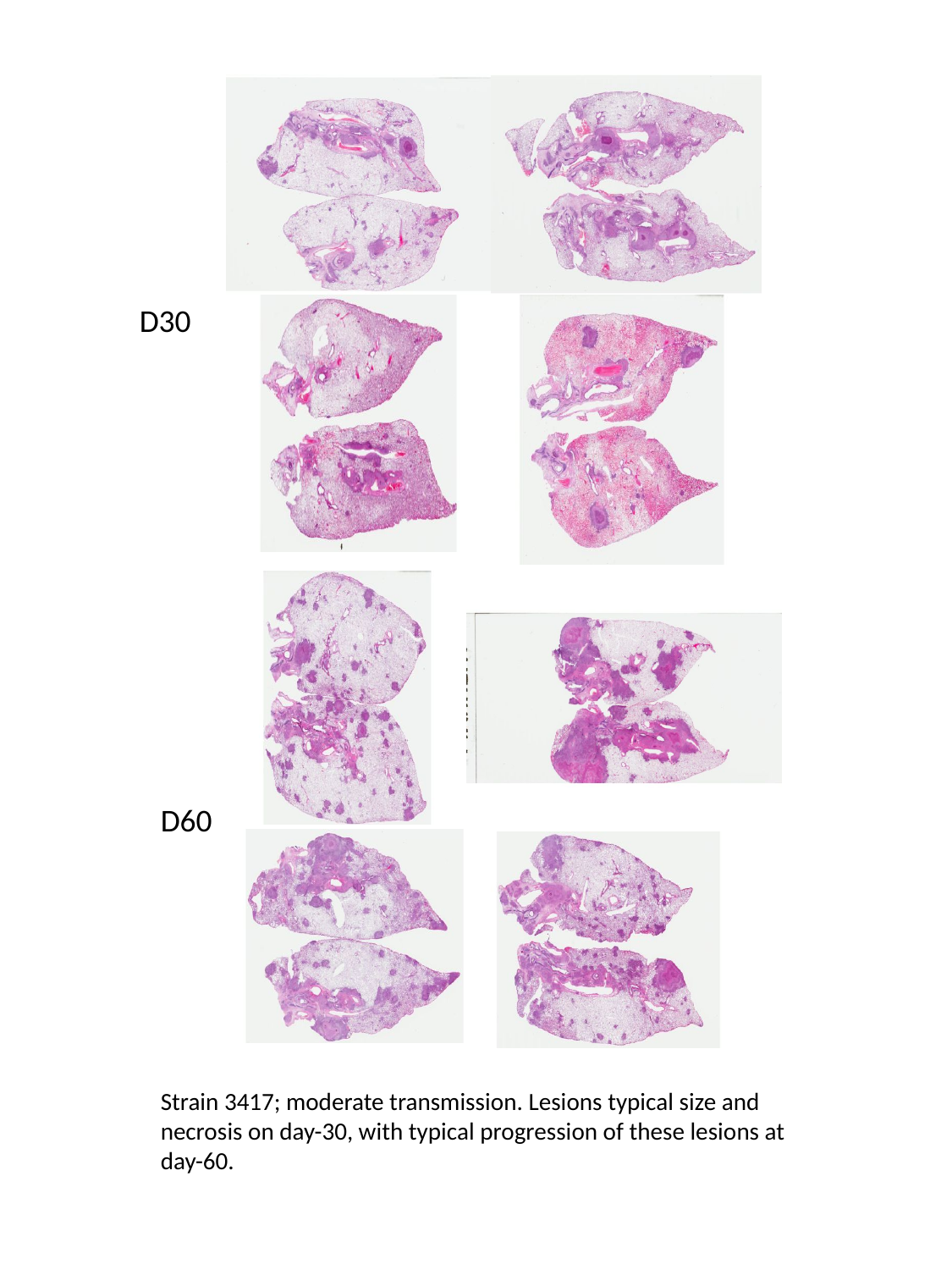

D30
D60
Strain 3417; moderate transmission. Lesions typical size and necrosis on day-30, with typical progression of these lesions at day-60.

## Slide 9
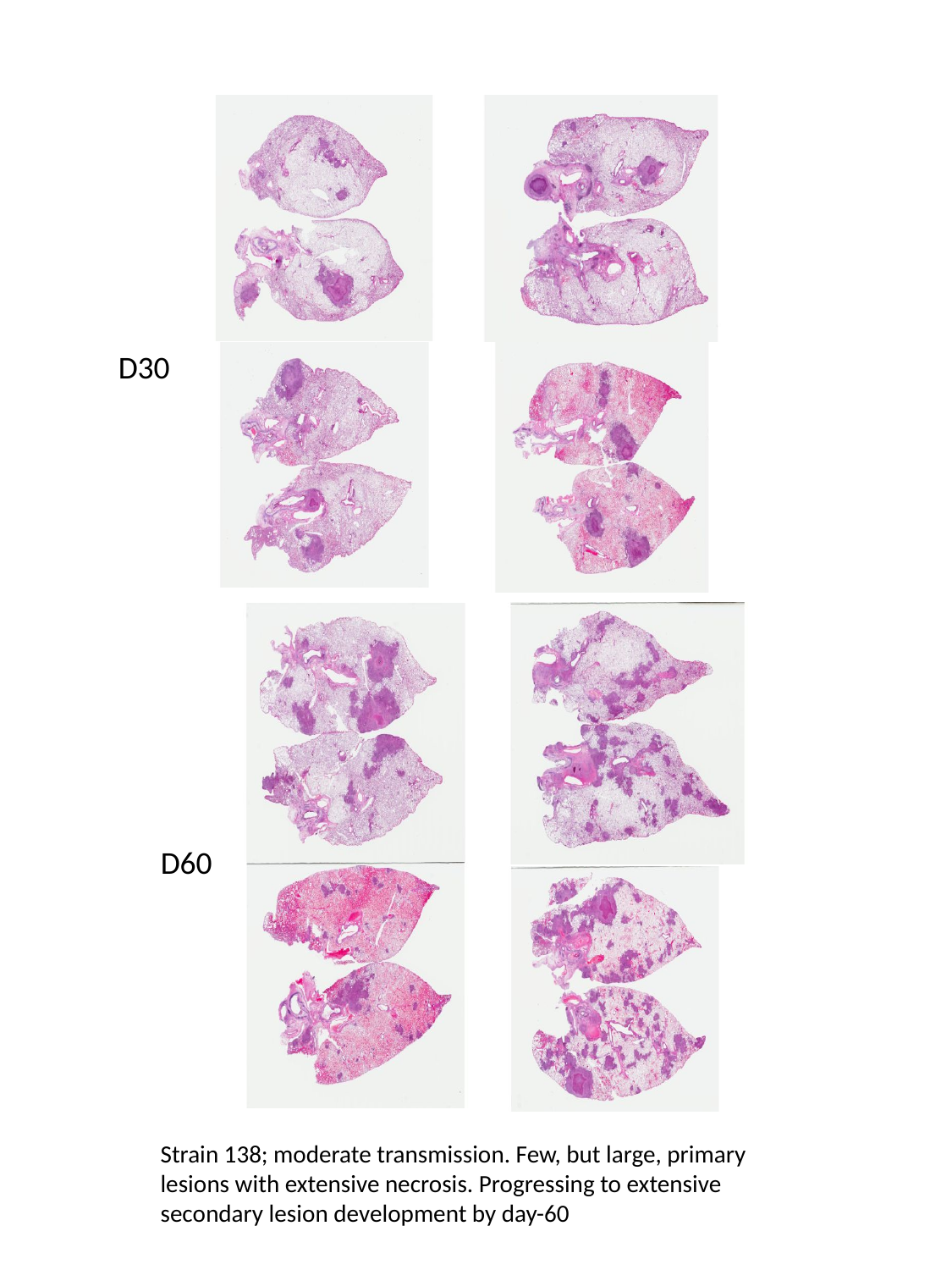

D30
D60
Strain 138; moderate transmission. Few, but large, primary lesions with extensive necrosis. Progressing to extensive secondary lesion development by day-60

## Slide 10
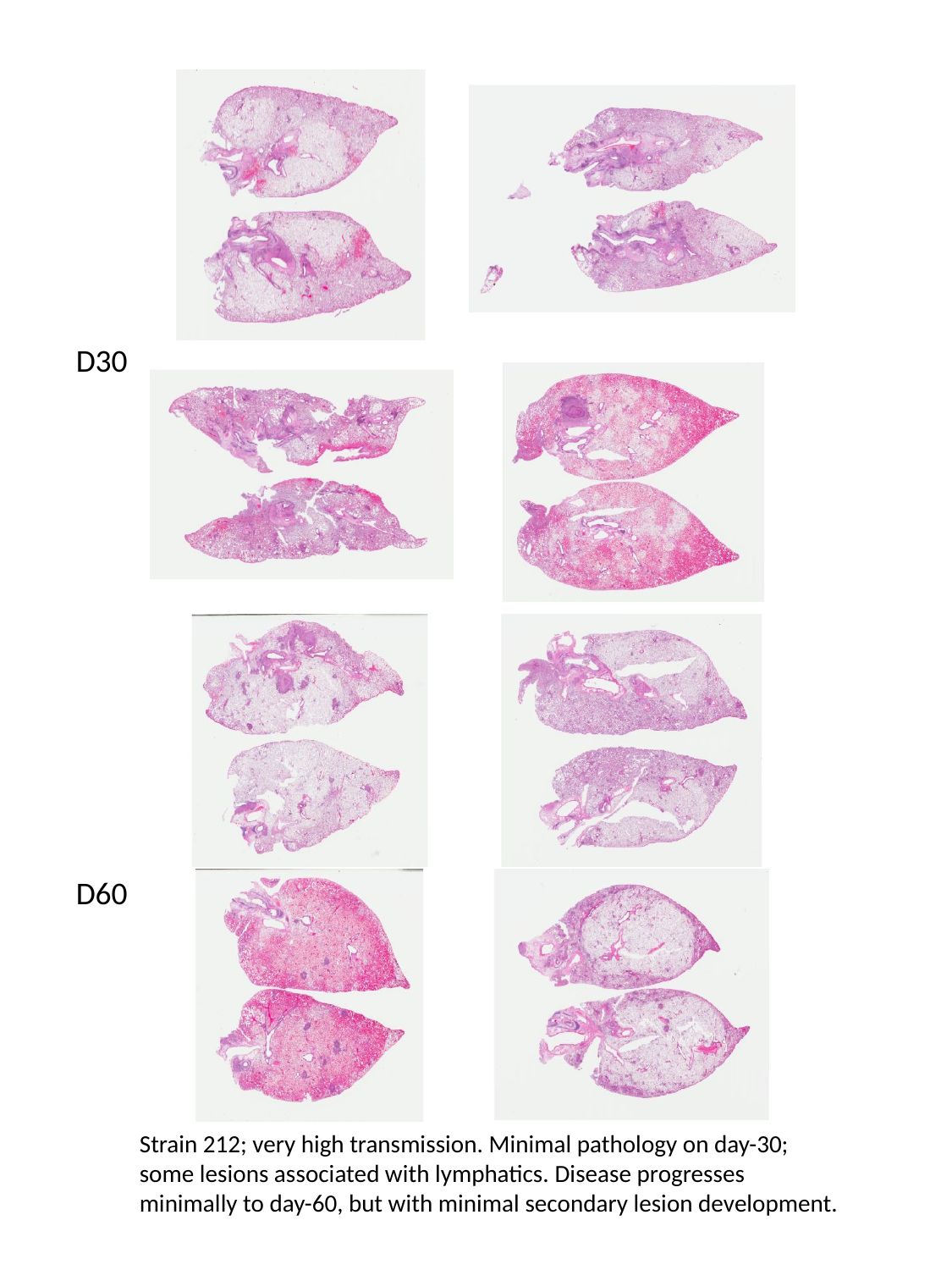

D30
D60
Strain 212; very high transmission. Minimal pathology on day-30; some lesions associated with lymphatics. Disease progresses minimally to day-60, but with minimal secondary lesion development.

## Slide 11
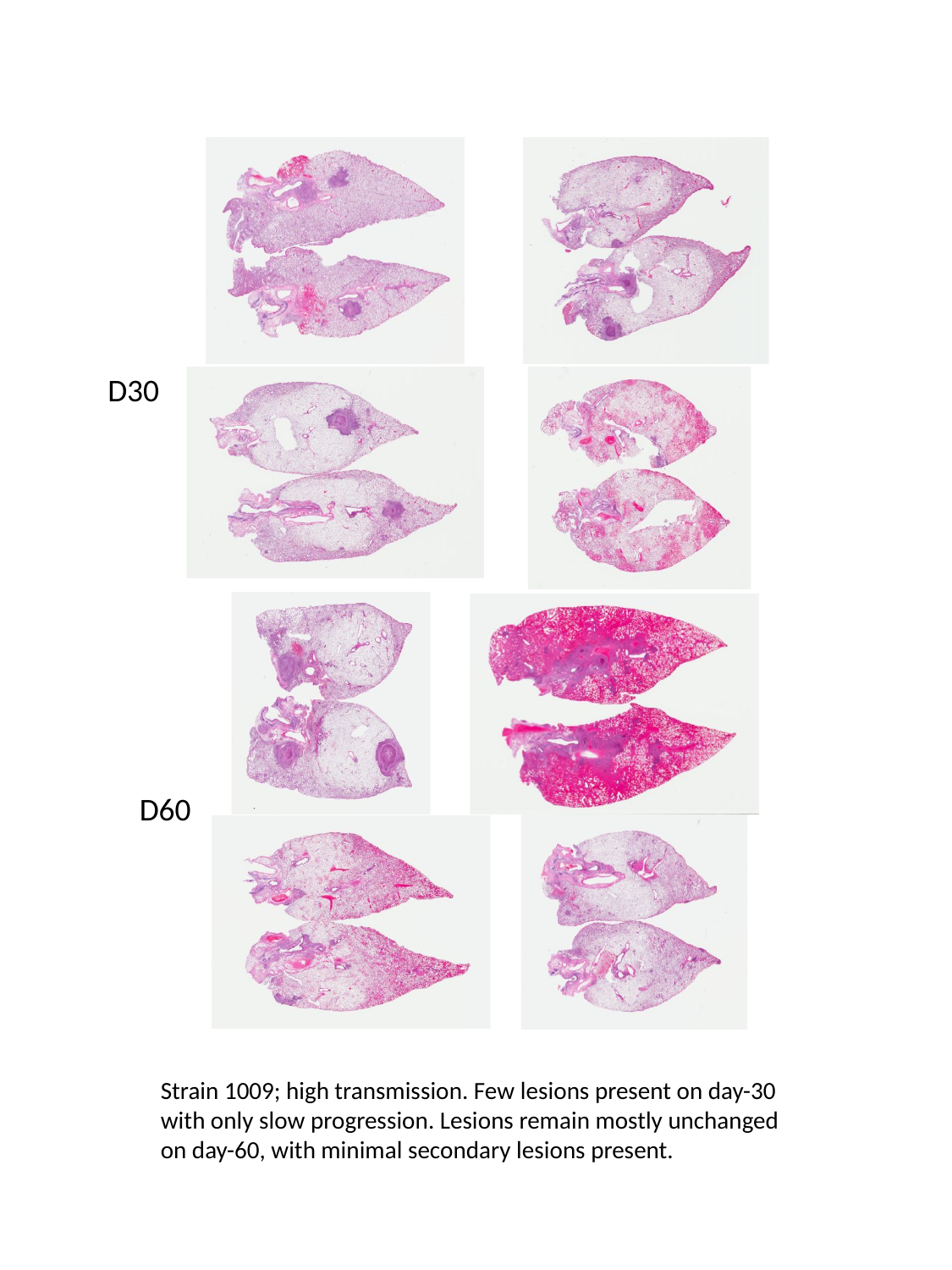

D30
D60
Strain 1009; high transmission. Few lesions present on day-30 with only slow progression. Lesions remain mostly unchanged on day-60, with minimal secondary lesions present.

## Slide 12
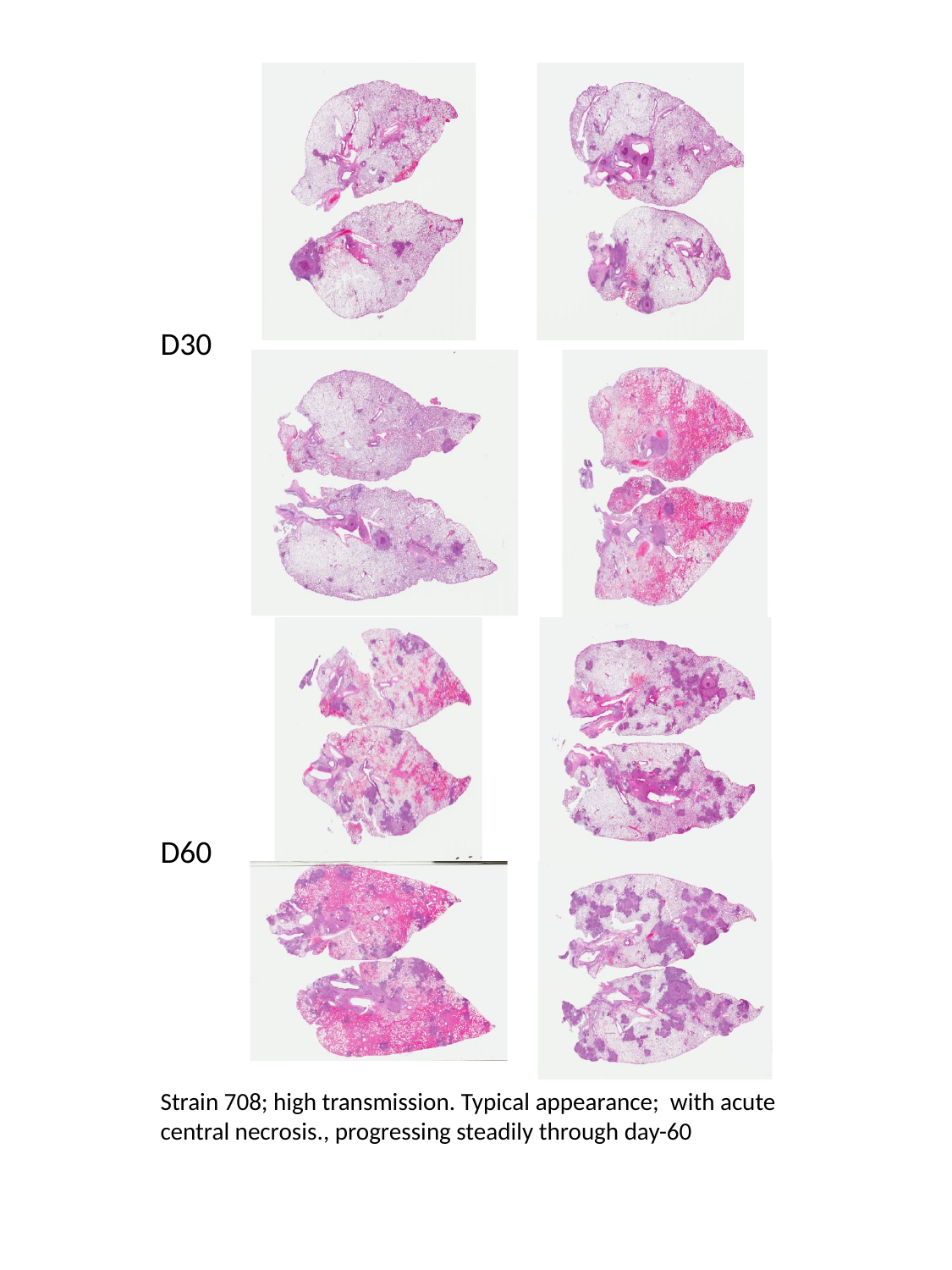

D30
D60
Strain 708; high transmission. Typical appearance; with acute central necrosis., progressing steadily through day-60
